# Enhancing Attitudes and Health Literacy Toward Dementia Through Dialogue Café: A Study Among Japanese Citizens and Healthcare Professionals

**DOI:** 10.7759/cureus.95803

**Published:** 2025-10-31

**Authors:** Daisuke Son, Toshichika Mitsuyama, Yuki Yonekura, Kazuhiro Nakayama

**Affiliations:** 1 Department of Community-Based Family Medicine, Faculty of Medicine, Tottori University, Yonago, JPN; 2 Department of Medical Education Studies, International Research Center for Medical Education, Graduate School of Medicine, The University of Tokyo, Tokyo, JPN; 3 General Home Care Clinic, Medical Corporation Kagayaki, Gifu, JPN; 4 Department of Nursing Informatics, Graduate School of Nursing Science, St. Luke's International University, Tokyo, JPN; 5 Department of Nursing Informatics, Graduate School of Nursing Science, St. Luke’s International University, Tokyo, JPN

**Keywords:** community-based intervention, dementia awareness, dialogue café, health literacy, public health education

## Abstract

Background: As global populations age, dementia poses a major public health challenge, necessitating community-based interventions to improve awareness and understanding. Dialogue Cafés, where healthcare professionals engage with local residents, offer a promising approach to enhancing public understanding and health literacy related to dementia. This study evaluates the effectiveness of Dialogue Café sessions in improving attitudes toward dementia and health literacy related to dementia comprehension among community citizens and healthcare/social professionals.

Methods: From October 2015 to February 2016, Dialogue Café sessions on dementia were conducted in six Japanese municipalities, involving 224 participants (217 completed questionnaires). These community-based educational cafés provided structured group discussions between healthcare professionals and citizens using the Dialogue Café approach. Attitude toward dementia and health literacy were measured at three time points: before (T1), immediately after (T2), and one month later (T3).

Results: The study included 202 participants (112 health professionals, 90 citizens). Health professionals’ Attitude Toward Dementia scale (AD) scores increased from 46.14 (±5.99) at T1 to 47.53 (±5.43) at T2 but slightly declined at T3 (46.74 ±5.56). Citizens showed a greater increase from 42.09 (±7.07) at T1 to 45.35 (±6.40) at T2, with a slight decrease at T3 (44.86 ±5.43). Health literacy improved in both groups, with sustained gains for citizens. Attitude changes were influenced by time, dementia course participation, and caregiving experience.

Conclusion: Dialogue Cafés effectively enhance dementia awareness and health literacy, particularly among the general public, supporting their broader application in community-based dementia education.

## Introduction

As global populations continue to age, dementia has become a major public health challenge, necessitating innovative, community-based interventions to improve awareness and understanding of this complex condition [1-3]. Dementia not only affects individuals but also profoundly impacts families and communities, leading to social isolation, caregiver burden, and stigma. Early education and public awareness are therefore essential to promoting timely help-seeking, improving quality of life, and building dementia-friendly communities. Dementia, a syndrome primarily affecting older adults and characterized by cognitive decline, places an increasing burden on healthcare systems worldwide [4]. As its prevalence rises alongside greater longevity, enhancing health literacy about aging and dementia among local residents is becoming increasingly critical.

One approach that has gained traction in health education is the use of Dialogue Cafés [5-7]. These interactive forums, where medical and welfare specialists engage with residents through thematic discussions, have been organized globally, demonstrating their broad appeal and potential scalability. Dialogue Cafés provide a platform for meaningful learning and engagement, potentially enhancing public health literacy, particularly in relation to aging and dementia [7].

Dialogue Cafés could serve as an effective mechanism for raising dementia awareness among local residents [8,9]. By fostering conversations between healthcare professionals and community members, they help bridge knowledge gaps and dispel misconceptions about dementia. Moreover, they offer an opportunity for bidirectional learning, allowing residents to gain insights from experts while enabling professionals to better understand local perceptions and concerns about dementia [10,11].

Despite their potential, empirical evidence supporting the effectiveness of Dialogue Cafés remains limited. This gap underscores the need for rigorous evaluation of such interventions. Therefore, this study aims to examine the effectiveness of Dialogue Cafés as a platform for health education, with a focus on dementia as the central theme. Through this study, we seek to determine whether Dialogue Cafés enhance public understanding of dementia and health literacy related to dementia comprehension among community citizens and healthcare/social professionals, thereby contributing to a more dementia-aware and dementia-literate society. The findings could provide valuable insights to inform future health education strategies and community-based interventions, particularly in the context of aging populations and the growing global challenge of dementia.

## Materials and methods

Dialogue café on dementia

From October 2015 to February 2016, Dialogue Café sessions on dementia were held in six municipalities across Japan, namely, Akita, Miyagi, Chiba, Tokyo, Hyogo, and Okinawa prefectures, applying the Dialogue Café approach as a structured community-based educational intervention. These sessions involved local residents as well as medical and welfare professionals. The Dialogue Cafés were conducted using the World Café method [12]. This method, developed by Juanita Brown and David Isaacs in 1995, was initially designed to facilitate discussions in business meetings. Each Dialogue Café session lasted approximately 90-120 minutes and was held once at each of the six sites, typically on weekends or weekday evenings to maximize participation. Participants sat in small groups (typically four to seven people per table) and, with guidance from a facilitator, periodically switched tables to exchange ideas on specific topics. Several rounds of discussion and reflection were conducted following the standard World Café format. Key points are recorded on table sheets, and after several rounds of discussion, participants return to their original tables to reflect on the insights gained. In this study, the Dialogue Café served as an educational intervention, and no qualitative data from the discussions were analyzed; all outcomes were quantitatively assessed through standardized questionnaires.

In this study’s dementia Dialogue Cafés, sessions began with experts providing a brief talk on dementia, sharing fundamental knowledge and personal narratives. This was followed by interactive discussions, where participants engaged in structured dialogues to deepen their understanding of dementia-related issues.

Participants and study design

This quasi-experimental pre-post study used three measurement time points: T1 (baseline, before the Dialogue Café), T2 (immediately after), and T3 (one-month follow-up). Participants were community citizens and healthcare/social professionals; no clinical staging of dementia was undertaken because the sample did not include patients with diagnosed dementia. Because this was a community-based educational intervention, no control group was included; changes in attitudes and health literacy were evaluated through repeated measures across the three time points.

A total of 224 individuals attended the Dialogue Cafés across six locations, and 217 (96.9%) completed the study questionnaire. These Cafés were originally organized as regular events in various locations. For this study, participants were invited to take part, and their agreement was confirmed through their questionnaire responses.

Questionnaires were administered at three time points: immediately before (T1), immediately after (T2), and one month after (T3) participation. The one-month follow-up questionnaire was distributed at the end of the Café session, along with a stamped return envelope, and was completed at home and mailed back.

The questionnaire included the following measures: Attitude Toward Dementia Scale (AD) (Japanese version, 15 items, four-point Likert scale) [13]; Communicative and Critical Health Literacy Scale (HL) (Japanese version, five items, five-point Likert scale) [14]; Demographic and background variables, including age, gender, household composition, occupation, experience caring for a family member with dementia, and prior participation in dementia-related courses.

Additionally, the one-month follow-up questionnaire included items assessing the AD and the HL scales to examine the sustainability of changes resulting from the intervention (Appendix A).

Both the AD [13] and the HL [14] are freely available for non-commercial academic use. 

Data analysis

Because this was a community-based educational intervention without a control group, the study was conducted as a quasi-experimental pre-post design using repeated measures across three time points. Statistical analyses focused on changes in scores rather than causal comparisons between groups.

To assess longitudinal changes in scores of AD and HL, generalized linear mixed models were used, incorporating repeated measures. Cases with missing values were handled using listwise deletion, which allowed analyses to be performed on complete cases for each variable. Separate models were constructed for health and social professionals (occupation 1) and citizens (occupation 2) to evaluate differences between occupational groups.

Dependent variables were the AD score and the HL score over time. Fixed effects included time, age, gender, household type, participation in dementia courses, dementia-family caregiving status, and HL change. Random effects included individual-level ID and region.

To evaluate the magnitude of change, Cohen’s d effect sizes were calculated for AD change and HL change. Effect sizes were interpreted as small (d = 0.2), moderate (d = 0.5), and large (d = 0.8).

Before conducting any statistical tests, we removed rows with missing values in key variables required for analysis. Specifically, when computing AD change and HL change, rows where either AD scores at T1 or T2 (or HL scores at T1 or T2) were missing were dropped. This brought the final sample size to a total of 202, with 112 health and social professionals and 90 citizens. In the generalized linear mixed model for AD and HL changes, cases with missing values in any of the predictor or outcome variables were excluded from the model (listwise deletion). The model was run separately for occupation 1 and occupation 2, and missing values in specific time points led to reduced sample sizes at T3.

Because the participants were community citizens and healthcare/social professionals rather than patients with dementia, variables such as the clinical stage of dementia or cognitive test scores were not applicable. Potential confounders, including age, gender, caregiving experience, and previous participation in dementia-related courses, were adjusted for in the generalized linear mixed models.

All analyses were conducted using Python 3.9 (Python Software Foundation (PSF), Beaverton, OR) (a community-developed open-source project led by Skipper Seabold and Josef Perktold, hosted on GitHub) and SciPy 1.8.0 (a community-developed open-source project under NumFOCUS, Austin, TX). A significance threshold of p < 0.05 was applied.

Ethical considerations

To protect participants' privacy and personal information, all questionnaires were anonymized. A request for study participation was included on the cover page of the questionnaire. Even when consent was obtained, we explicitly stated in writing that participation in this study was voluntary and that respondents could choose whether to answer each question. This study was approved by the Ethics Review Committee of the University of Tokyo School of Medicine (approval no. 10954).

## Results

Participant characteristics

Table 1 summarizes the demographics of the study participants, including 202 individuals: 112 health and social professionals and 90 citizens. The mean age of the professionals was 45.56 years (range: 21-75), while the citizens had a higher mean age of 58.28 years (range: 25-92). Among the professionals, 25.9% were male and 74.1% were female, while among the citizens, 57.8% were male and 42.2% were female. The percentage of single-person households was similar between the two groups, at 14.3% for professionals and 15.6% for citizens.

**Table 1:** Demographics of the study participants † AD represents the Attitude Toward Dementia Scale [13]; ‡ HL represents the Communicative and Critical Health Literacy Scale [14]

Variable	Health and social professionals	Citizens
N	112	90
Age (years) (mean ± SD, range)	45.56 ± 10.88 [21–75]	58.28 ± 14.25 [25–92]
Gender (male)	29 (25.9%)	52 (57.8%)
Single-person household	16 (14.3%)	14 (15.6%)
Participation in dementia-related courses	90 (80.4%)	44 (48.9%)
Experience caring for a family member with dementia	44 (39.3%)	27 (30.0%)
Baseline AD score (mean ± SD)^ †^	46.14 ± 5.99	42.09 ± 7.07
Baseline HL score (mean ± SD)^‡^	18.38 ± 3.33	18.09 ± 4.34
Location		
Akita City, Akita	37 (33.0%)	15 (16.7%)
Sendai City, Miyagi	8 (7.1%)	11 (12.2%)
Choshi City, Chiba	31 (27.7%)	7 (7.8%)
Fuchu City, Tokyo	4 (3.6%)	17 (18.9%)
Himeji City, Hyogo	25 (22.3%)	14 (15.6%)
Minamidaito Island, Okinawa	7 (6.3%)	26 (28.9%)

A significantly higher proportion of health and social professionals (80.4%) had participated in dementia-related courses compared to citizens (48.9%). Additionally, 39.3% of professionals had experience caring for a family member with dementia, versus 30.0% of citizens. The baseline AD scores were higher for professionals (46.14) than for citizens (42.09), while the baseline HL scores were similar (18.38 for professionals and 18.09 for citizens).

Participants were from various locations, with the largest proportions of professionals from Akita City (33.0%) and citizens from Minamidaito Island (28.9%).

Changes in attitudes toward dementia

**Figure 1:**
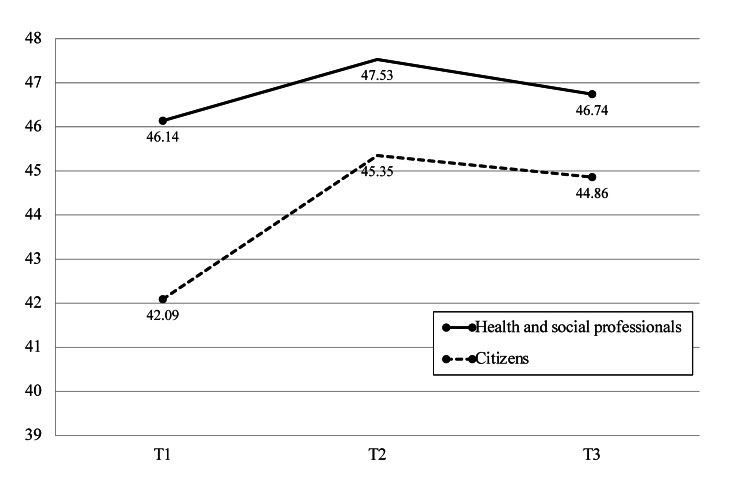
Change in Attitude Toward Dementia Scale scores before and after Dialogue Café Source: [13]

Table 2 and Figure 1 present the changes in the AD scores before and after participation in the Dialogue Café, comparing health and social professionals and citizens.

**Table 2:** Change of scores of Attitude Toward Dementia Scale before and after Dialogue Café † Sample size (N) varies across time points due to participant attrition; ‡ Effect sizes (Cohen’s d) were calculated for the change from T1 to each corresponding time point. Source: [13]

Time point	N^†^	Mean ± SD	Effect size^‡^	N	Mean ± SD	Effect size
	Health and social professionals			Citizens		
T1	112	46.14 ± 5.99	-	90	42.09 ± 7.07	-
T2	112	47.53 ± 5.43	0.349	90	45.35 ± 6.40	0.622
T3	54	46.74 ± 5.56	0.279	53	44.86 ± 5.43	0.526

At T1, the mean AD score for health and social professionals was 46.14 (± 5.99), while citizens had a lower mean score of 42.09 (± 7.07). At T2, immediately following the Dialogue Café, both groups showed an increase in their AD scores. Health and social professionals had a mean score of 47.53 (± 5.43), with a small effect size of 0.349, indicating a modest improvement. Citizens demonstrated a larger increase, with a mean score of 45.35 (± 6.40) and an effect size of 0.622, reflecting a more substantial change.

At T3, one month after participation, the AD scores slightly decreased compared to T2, with health and social professionals scoring 46.74 (± 5.56) and citizens scoring 44.86 (± 5.43). The effect sizes from T1 to T3 were 0.279 for professionals and 0.526 for citizens, indicating modest but continued improvements, with a more noticeable effect observed in citizens.

Changes in health literacy

**Figure 2:**
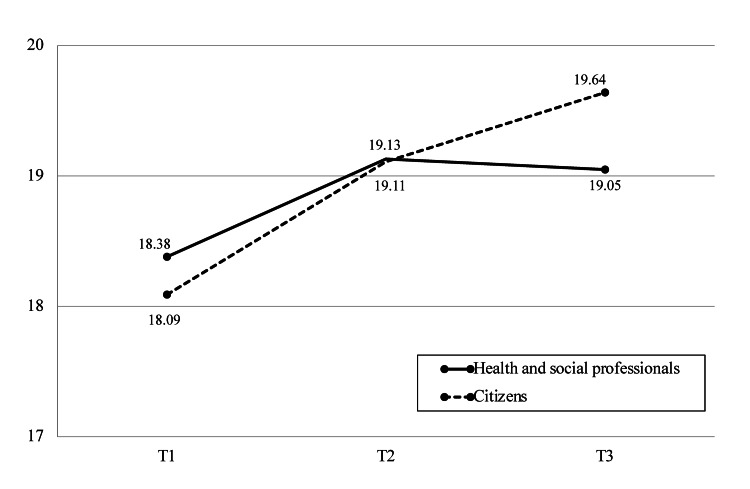
Changes in Health Literacy Scale scores before and after Dialogue Café Source: [14]

Table 3 and Figure 2 present the changes in HL scores before and after participation in the Dialogue Café, comparing health and social professionals and citizens.

**Table 3:** Change in Health Literacy Scale scores before and after Dialogue Café † Sample size (N) varies across time points due to participant attrition; ‡ Effect sizes (Cohen’s d) were calculated for the change from T1 to each corresponding time point. Source: [14]

Time point	N^†^	Mean ± SD	Effect size^‡^	N	Mean ± SD	Effect size
	Health and social professionals			Citizens		
T1	112	18.38 ± 3.33	-	90	18.09 ± 4.34	-
T2	112	19.13 ± 3.44	0.305	90	19.11 ± 3.70	0.257
T3	55	19.05 ± 3.70	0.084	52	19.64 ± 3.65	0.267

At T1, the mean HL score for health and social professionals was 18.38 (± 3.33), while citizens had a slightly lower mean score of 18.09 (± 4.34). Following the Dialogue Café, both groups showed an increase in HL scores at T2. Health and social professionals had a mean score of 19.13 (± 3.44), with a small effect size of 0.305, indicating a modest improvement. Citizens showed a similar increase, with a mean score of 19.11 (± 3.70) and an effect size of 0.257.

At T3, one month after participation, there was a slight decrease in scores for health and social professionals, with a mean score of 19.05 (± 3.70) and a small effect size of 0.084. In contrast, citizens showed a further increase in their HL score, reaching 19.64 (± 3.65), with an effect size of 0.267, indicating a modest continued improvement.

Factors associated with changes in attitudes toward Dementia in health and social professionals

Table 4 presents the results of the generalized linear mixed model analyzing the changes in the AD scores among health and social professionals (N = 112).

**Table 4:** Generalized linear mixed model results with changes in Attitude Toward Dementia Scale [13] in health and social professionals Note: p < 0.05 (*) indicating statistically significant difference; † Health Literacy Scale (HL) score change represents the pre-to-post café change (T2 - T1) in HL score. [14]; ‡ Group variance indicates variability in scale scores across individuals. § Time variance indicates variability in scale scores over time in repeated measures.

	Coefficient	Standard Error	z	P-value
Intercept	44.775	2.800	15.992	<0.001
Time	0.635	0.251	2.525	0.012*
Age	0.001	0.050	0.009	0.993
Gender	0.126	1.214	0.104	0.917
Single-person household	0.055	1.480	0.037	0.970
Participation in dementia-related courses	1.520	1.320	1.151	0.250
Experience caring for a family member with dementia	-0.767	1.064	-0.721	0.471
Health literacy score change^†^	-0.247	0.207	-1.190	0.234
Group variance^‡^	29.462	3.423		
Group × time covariance	-1.475	0.835		
Time variance^§^	0.074	0.318		

The intercept of the model was 44.775 (standard error (SE) = 2.800), indicating the baseline AD score at T1. Time was a significant predictor, with a coefficient of 0.635 (SE = 0.251, z = 2.525, p = 0.012), suggesting that the AD scores significantly increased from T1 to T2. In contrast, age, gender, single-person household status, participation in dementia-related courses, and experience caring for a family member with dementia were not statistically significant predictors of changes in AD scores, as their p-values were all above 0.05. The change in HL scores, represented by the pre-to-post Dialogue Café change (T2 - T1), also did not significantly predict changes in AD scores, with a coefficient of -0.247 (SE = 0.207, z = -1.190, p = 0.234). Additionally, the group variance (29.462) indicates substantial variability in AD scores across individuals, while the time variance (0.074) suggests small variability in AD scores over time.

These results indicate that, among health and social professionals, time significantly influenced changes in attitudes toward dementia, but other demographic and experience-related factors, including health literacy changes, did not show significant effects.

Factors associated with changes in attitudes toward dementia in citizens

Table 5 presents the results of the generalized linear mixed model analyzing changes in the AD scores among citizens (N = 90).

**Table 5:** Generalized linear mixed model results with changes in Attitude Toward Dementia Scale in citizens Note: p < 0.05 (*) and p < 0.01 (**), indicating statistically significant difference. In this model, the double asterisk (**) applies to the Time variable only; Age was not a significant predictor. † Health literacy score change represents the pre-to-post café change (T2 - T1) in health literacy scale. [14] ‡ Group variance indicates variability in scale scores across individuals. § Time variance indicates variability in scale scores over time in repeated measures. Source: [13]

Variable	Coefficient	Standard Error	z	P-value
Intercept	40.114	3.133	12.805	<0.001
Time	1.595	0.322	4.958	<0.001**
Age	-0.044	0.046	-0.966	0.334
Gender	0.654	1.235	0.529	0.597
Single-person household	1.092	1.735	0.629	0.529
Participation in dementia-related courses	2.820	1.270	2.220	0.026*
Experience caring for a family member with dementia	3.187	1.391	2.292	0.022*
Health literacy score change^†^	0.095	0.159	0.597	0.551
Group variance^‡^	38.572	4.691		
Group × time covariance	-3.171	1.270		
Time variance^§^	0.279	0.532		

The intercept of the model was 40.114 (SE = 3.133), reflecting the baseline AD score at T1. Time was a significant predictor, with a coefficient of 1.595 (SE = 0.322, z = 4.958, p < 0.001), indicating a significant increase in AD scores from T1 to T2. This suggests that participation in the Dialogue Café led to a substantial positive shift in attitudes toward dementia among citizens.

Age was not a significant predictor of changes in AD scores (coefficient = -0.044, SE = 0.046, z = -0.966, p = 0.334). However, participation in dementia-related courses and experience caring for a family member with dementia were both significant predictors, with coefficients of 2.820 (SE = 1.270, z = 2.220, p = 0.026) and 3.187 (SE = 1.391, z = 2.292, p = 0.022), respectively, suggesting that these factors were associated with greater improvements in attitudes toward dementia. The change in HL scores (pre-to-post Dialogue Café change) was not a significant predictor, with a coefficient of 0.095 (SE = 0.159, z = 0.597, p = 0.551).

The group variance (38.572) suggests considerable variability in AD scores across individuals, while the time variance (0.279) indicates small variability in scores over time.

Overall, these results demonstrate that time, participation in dementia-related courses, and experience caring for a family member with dementia were significant predictors of changes in attitudes toward dementia among citizens.

## Discussion

This study aimed to evaluate the effectiveness of Dialogue Cafés as a community-based intervention for improving dementia awareness and health literacy. The results indicate that participation in the Dialogue Cafés led to significant improvements in attitudes toward dementia and health literacy among both health and social professionals and citizens, with some notable differences between the two groups. Dementia Cafés in Japan have shown promise as a community resource for individuals with dementia, with participants preferring frequent meetings and activities [10,11]. This aligns with our study, highlighting the effectiveness of community-based, interactive approaches in improving dementia awareness.

The most significant finding is the increase in AD scores observed immediately after the Dialogue Café (T2) for both groups. Health and social professionals demonstrated a modest improvement, while citizens experienced a more substantial increase in their AD scores. This may reflect the differing baseline knowledge and familiarity with dementia between the two groups, as health and social professionals generally had more experience and prior knowledge of dementia, which could have moderated their responses to the intervention. The sustained improvements in attitudes one month later (T3) suggest that the Dialogue Cafés had a lasting impact on participants' perceptions of dementia, particularly among citizens. Existing studies show that specialist dementia training for healthcare professionals can improve attitudes and knowledge with long-term effects [15]. However, they focused only on professionals. This study is the first to include both healthcare professionals and citizens, demonstrating positive attitude changes toward dementia.

In terms of HL, both groups showed a modest increase in scores immediately after the Dialogue Café (T2), with citizens showing a slightly higher effect size than professionals. Interestingly, health and social professionals experienced a small decline in HL scores at T3, while citizens continued to show a slight improvement. This discrepancy may be due to the fact that citizens started with lower baseline health literacy scores, which could have allowed for more noticeable improvements in a shorter period. In contrast, health and social professionals, who may have had higher initial levels of health literacy, may have experienced a plateau in their learning after the intervention.

The generalized linear mixed model analysis provided further insights into the factors associated with changes in AD scores. Among health and social professionals, the only significant predictor of change was time, which reflects the overall positive impact of the intervention. Other variables, such as age, gender, participation in dementia-related courses, and experience caring for a family member with dementia, were not significant predictors of attitude change. This finding suggests that, for professionals, the structured nature of the Dialogue Café itself, rather than prior knowledge or personal experience, played a key role in enhancing dementia awareness.

In contrast, among citizens, several factors were found to significantly predict changes in AD scores. Participation in dementia-related courses and experience caring for a family member with dementia were associated with larger improvements in attitudes toward dementia. This suggests that the Dialogue Café was particularly effective for citizens who had a personal or educational connection to dementia, emphasizing the importance of tailored interventions that build upon participants' existing knowledge and experiences. Given that health literacy is crucial for informed decision-making in dementia care [11,16-18], these findings underscore the importance of interactive, discussion-based education models. A previous study has shown that Dialogue Cafés can improve health literacy among citizens; however, it was cross-sectional and did not measure sustained changes [7]. A qualitative study on dementia patients and caregivers highlights the importance of improving health literacy through self-initiative and community resources [19]. Similarly, our research demonstrates that Dialogue Cafés can enhance health literacy and dementia-related knowledge, emphasizing the value of community-based interventions in supporting individuals with dementia.

These findings have important implications for the design and implementation of community-based health education interventions. The Dialogue Café method proved to be an effective platform for increasing dementia awareness and health literacy, particularly for citizens who may have had less prior exposure to dementia-related topics. Furthermore, the sustained improvements observed in citizens' AD scores and HL scores highlight the potential for Dialogue Cafés to foster long-term changes in attitudes and knowledge.

One limitation of the study is the attrition rate, particularly at the one-month follow-up (T3), which may have introduced bias in the results. Because missing data were handled using listwise deletion, the impact of attrition may have been amplified, potentially introducing additional bias. In addition, because this quasi-experimental study did not include a control group and evaluated only short-term outcomes, causal inferences and long-term effects should be interpreted with caution. Future research should consider strategies to minimize participant drop-out and explore the long-term impact of Dialogue Cafés on dementia awareness. Additionally, future studies could investigate the specific mechanisms through which Dialogue Cafés influence health literacy and attitudes toward dementia, as well as the potential for these interventions to be scaled to larger populations. Furthermore, because the participants were community citizens and healthcare/social professionals rather than patients with dementia, clinical staging of dementia was not assessed, which may limit comparisons with patient-based studies. In addition, demographic diversity (participants aged between 20 to 70 years) and unmeasured lifestyle factors such as smoking, alcohol consumption, or medication use could have influenced the results. Future studies should examine whether these variables affect the impact of Dialogue Café interventions. Finally, the relatively small sample size and the self-selected nature of the participants may have introduced selection bias and limited the generalizability of the findings to other cultural or community contexts.

## Conclusions

This study provides empirical evidence supporting the effectiveness of Dialogue Cafés as a community-based intervention for improving dementia awareness and health literacy. The findings suggest that Dialogue Cafés can play a crucial role in enhancing public understanding of dementia, particularly among citizens, and offer valuable insights for the development of future health education programs aimed at addressing the challenges posed by an aging population and the increasing prevalence of dementia.

Moreover, the study highlights the potential of interactive, discussion-based approaches to bridge the gap between healthcare professionals and citizens, fostering mutual learning and social inclusion. Future research should further explore the long-term sustainability and scalability of Dialogue Cafés and evaluate their impact on behavioral and community-level outcomes. Such efforts could inform the design of participatory public health strategies that strengthen community resilience in the face of demographic change.

## Figures and Tables

**Figure 3:**
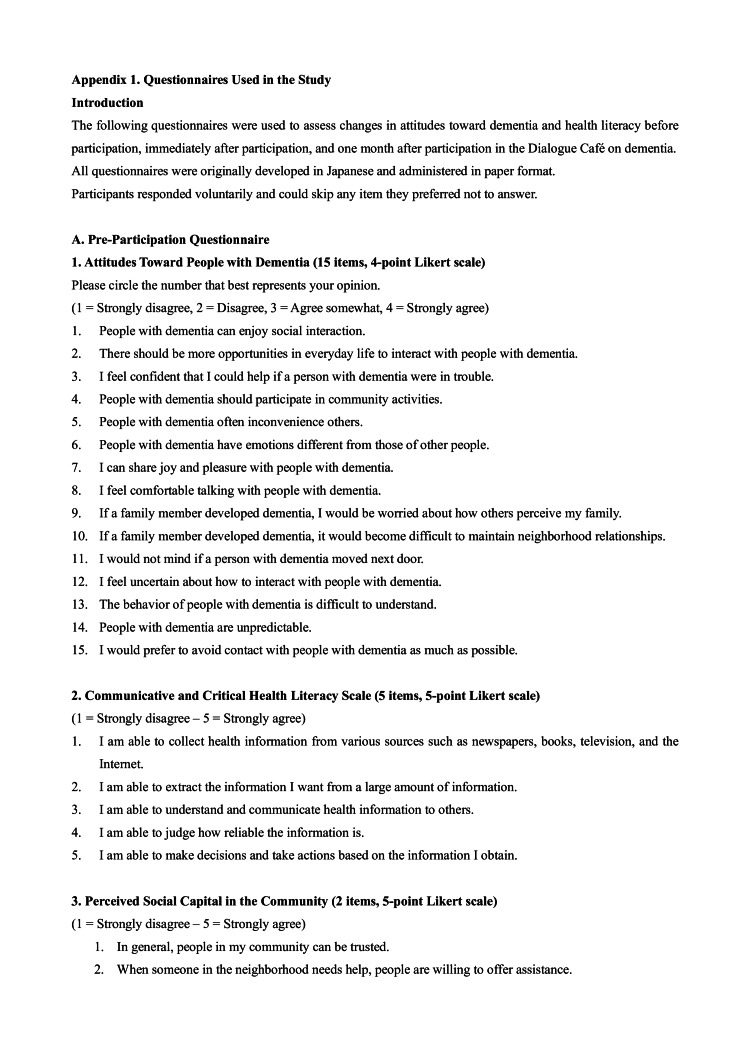
The one-month follow-up questionnaire: part 1

**Figure 4:**
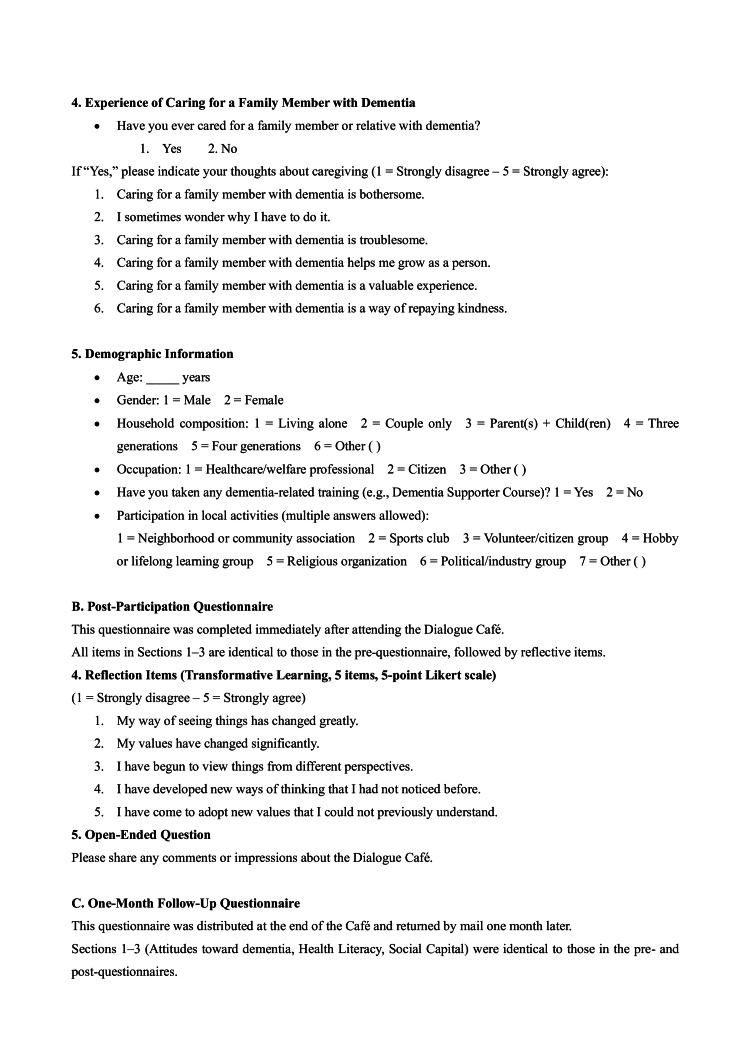
The one-month follow-up questionnaire: part 2

**Figure 5:**
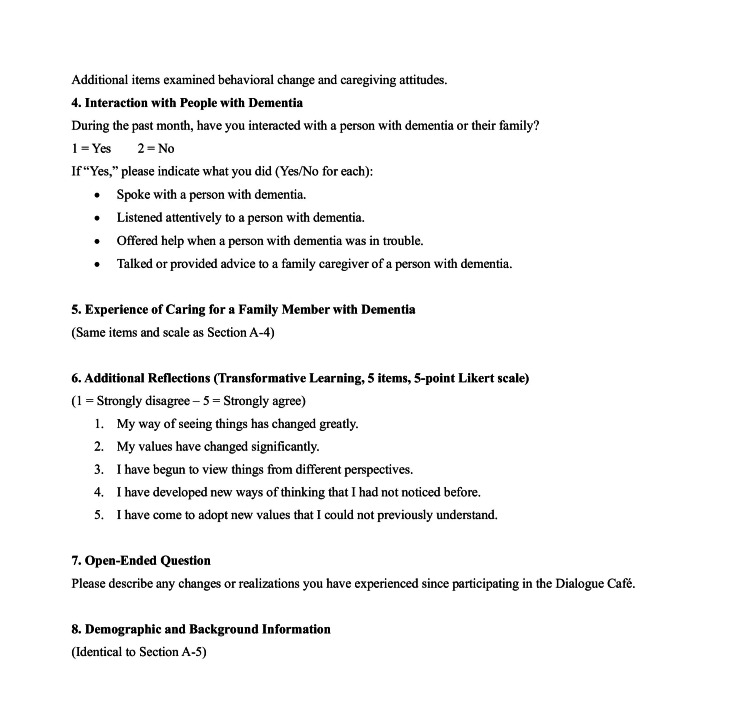
The one-month follow-up questionnaire: part 3

## Appendices

Appendix A
